# Antibiotic-free selection in E. coli: new considerations for optimal design and improved production

**DOI:** 10.1186/1475-2859-9-65

**Published:** 2010-09-07

**Authors:** Isabelle Peubez, Nicolas Chaudet, Charlotte Mignon, Géraldine Hild, Stéphanie Husson, Virginie Courtois, Karelle De Luca, Denis Speck, Régis Sodoyer

**Affiliations:** 1 Sanofi pasteur: Research Department, 1541 av. Marcel Mérieux 69280 Marcy l'Etoile France; 2 Sanofi pasteur: Process Development, 1541 av. Marcel Mérieux 69280 Marcy l'Etoile France

## Abstract

**Background:**

The increasing regulatory requirements to which biological agents are subjected will have a great impact in the field of industrial protein expression and production. There is an expectation that in a near future, there may be "zero tolerance" towards antibiotic-based selection and production systems. Besides the antibiotic itself, the antibiotic resistance gene is an important consideration. The complete absence of antibiotic-resistance gene being the only way to ensure that there is no propagation in the environment or transfer of resistance to pathogenic strains.

**Results:**

In a first step, we have designed a series of vectors, containing a stabilization element allowing a complete elimination of antibiotics during fermentation. Vectors were further improved in order to include alternative selection means such as the well known poison/antidote stabilization system. Eventually we propose an elegant positive pressure of selection ensuring the elimination of the antibiotic-resistance gene through homologous recombination. In addition, we have shown that the presence of an antibiotic resistance gene can indirectly reduce the amount of expressed protein, since even in absence of selection pressure the gene would be transcribed and account for an additional stress for the host during the fermentation process.

**Conclusions:**

We propose a general strategy combining plasmid stabilization and antibiotic-free selection. The proposed host/vector system, completely devoid of antibiotic resistance gene at the end of construction, has the additional advantage of improving recombinant protein expression and/or plasmid recovery.

## Background

Antibiotics are commonly used during bacterial fermentation, and the vast majority of expression vectors contain antibiotic resistance genes as selection markers. The degree of acceptance of these markers is both antibiotic and context dependent: Kanamycin and, to a lesser extent, Tetracycline, are still acceptable to the health authorities. In contrast the use of β-lactams, often associated with allergic responses, is strictly prohibited. Requirement can also be variable according to the nature of the therapeutic product, the presence of an antibiotic resistance gene, tolerated on a vector expressing a recombinant biopharmaceutical, will be totally undesirable on a gene therapy plasmid.

Different strategies are currently used and are described below.

### Post-segregational killing

Post-segregational killing is a mechanism by which plasmids are stably maintained by expressing a gene product that would be toxic to cells becoming plasmid-free upon division.

The most representative example of post-segregational killing of plasmid-free cells is the *Hok/Sok *system described for the first time in 1986. The translation of the *Hok *(host killing) messenger, encoding a toxin lethal to the bacteria, is completely blocked by the anti-messenger *Sok *(suppression of killing). In the absence of plasmid, *Sok*, which is less stable than *Hok*, is lost first, allowing the translation of the *Hok *mRNA and expression of the toxin lethal to the cell [1].

Other examples of the mechanism known as post-segregational killing have been reported such as plasmids carrying type II restriction modification (RM) gene complexes inhibiting the propagation of a cell population following chromosome breakage [2].

### Essential gene complementation

#### • The example of dapD

The most common way to achieve selection in the absence of antibiotics is via complementation of an essential gene making use of an expression vector in a strain with a defect or inhibited expression of the same essential gene. The *dapD *gene, which has a role in the Lysine biosynthetic pathway as well as cell wall assembly, has been selected as a preferred candidate by several authors, knowing that mutations in the dap pathway are lethal [3]. The limitations, in that case, are the intrinsic difficulty in construction of a *dapD *- mutant strain and dependence towards defined culture media composition.

#### • *dapD *and repressor titration

A more elaborated strategy still based on *dapD *was described: the so-called "operator repressor titration for antibiotic-free plasmid maintenance", proposed by Cobra Therapeutics, is a model in which the plasmid loss induces the downregulation of the essential *dapD *gene, and thus bacterial death [4].

#### • Translation initiation factor infA

As alternatives to *dapD*, other essential genes have been targeted for the same purpose, particularly *infA *coding for a translation initiation factor [5]. The authors show that the system is tightly regulated and that no cross-feeding effect is observed since initiation factors released into the media from lyzed cells are not absorbed by plasmid-free cells.

#### • Amino-acid auxotrophy complementation

A proline-auxotrophic K12 strain obtained through chromosomal *pro*BA gene deletion is described [6] for the expression of antibody Fab fragment. In that particular case the plasmid-mediated complementation is used as a second selection mechanism to completely abolish plasmid loss during fermentation.

According to the same principle an *E. coli *M15-derivated glycine-auxotrophic strain has been constructed and shown to produce comparable amounts of recombinant protein as a conventional system [7].

#### • The murA gene and RNA/RNA interaction

The principle of down regulating an essential gene upon plasmid loss has been exploited in a very original way for the design of new vectors for gene therapy [8,9]. The expression of the essential gene *murA *encoding an enzyme essential for the biosynthesis of cell wall is under control of the Tet repressor, TetR expression is inhibited by an RNA-RNA antisense interaction with RNAI derived from plasmid origin of replication *ColE1*. The major advantage of this system is that no additional sequence is required on the plasmid.

#### • RNA-OUT based antibiotic-free selection system

In a recent paper [10], a RNA based selectable marker, not restricted to *ColE1 *containing vectors is described. Briefly, a counter-selectable marker (sacB) levansucrase from Bacillus subtilis, under control of the RNA-IN promoter is integrated into the bacterial chromosome induces cell death in presence of sucrose.

Plasmid maintenance is ensured by the presence of the plasmid-borne regulator RNA-OUT anti-messenger acting as a down regulator of the expression of levansucrase.

### Plasmid selection using an endogenous essential gene marker

A potential drawback of essential gene complementation is the requirement of engineering mutant host strains and using specifically defined media. The *fabl*-triclosan model system recently proposed is based on the over-expression of a host essential gene in presence of a chemical inhibitor of its product [11].

#### • Mutual dependence: the pCOR system

Other systems such as pCOR, based on the complementation of an amber mutation, and conditional origin of replication have also been established. The original feature of the model is that an additional degree of refinement was introduced, since the dependence created between the host and the vector has become bilateral. Nevertheless, the requirement for a minimal medium for culture means these systems are more likely to be used for DNA production rather than recombinant protein over expression [12].

### Poison/Antidote selection

The original "Separate-component-stabilization system for protein and DNA production without the use of antibiotics" has recently been proposed by Szpirer *et al *[13]. The selection is based on the following elements:

- Gene *ccdB *(the poison), inserted into the bacterial genome of the *E. coli *strain BL21λ (DE3) and encoding a stable protein (100 aa), binding gyrase, an essential element for cell division. Upon binding gyrase, the *ccdB *gene product impairs DNA replication and induces cell death.

- Gene *ccdA *(the antidote), plasmid-born, encodes an instable protein (90 aa) under control of the *mob *promoter, acting as a natural inhibitor of ccdB.

It has been shown that after 20 generations on a non selective medium 100% of the bacteria still contain the plasmid. Two hours after induction, the plasmid is still present into all bacteria, which is not the case with a standard pET/BL21λDE3 system.

The various complementation-based expression systems or more generally antibiotic-free systems have the common drawback of being strain dependant, since genetic knockout or modification of an essential gene is not easily transferable from one strain to another and has to be done independently for each strain. Strain modification by chromosomal mutagenesis is still a tedious approach, notwithstanding the existence of technologies making use of counter-selectable vectors or PCR amplified fragments [14-16].

#### Plasmid stabilisation

A second aspect to be considered during the expression of a recombinant biopharmaceutical is the plasmid stabilisation that can be achieved by insertion of a genetic element such as the *cer *locus, which allows stable inheritance of *ColE1 *and related plasmids by preventing the runaway accumulation of multimers known as "dimer catastrophe" [17,18]. Multimer resolution is achieved through action of the XerCD site-specific recombinase at the *cer *site. Cloning of the *cer *locus into various expression vectors has been extensively documented and the proof of principle largely established in high-cell density cultures [19].

Here, we report a new strategy to overcome the need for antibiotics in the production process, including increasing plasmid stability and maintenance during fermentation as well as the use of alternative, "antibiotic-free" selection markers.

## Results and Discussion

### First step: Plasmid stabilisation

Plasmid stabilisation and increased maintenance during a fermentation process can be considered as a first step towards antibiotic starvation. Genetic elements allowing plasmid maintenance during cell division and limiting the probability of plasmid loss over generations should be considered.

In a first series of experiments the stabilisation effect of the *cer *locus was demonstrated during the expression of different recombinant antigens from *Helicobacter pylori*, at laboratory scale in shake flasks.

Two vectors, based on pET28 backbone, expressing the AlpA or Urease antigens from *Helicobacter pylori *have been modified in order to include a DNA fragment containing the *cer *locus in both orientations as compared to the expression cassette. From a technical point of view, the construct was done by ligation between two PCR fragments containing respectively the *cer *fragment and the entire pET28 plasmid devoid of the F1 origin. The assembly was made possible by the complementarity of two newly created unique restriction site brought by the PCR primers used during the amplification steps.

The readout of the stability testing experiment was a simple counting, after plating on selective medium, of bacterial colonies still containing the plasmid at different times of culture in absence of antibiotic selection.

As shown in tables 1 and 2, the plasmid maintenance is dramatically increased due to the presence of the *cer *fragment, whatever the antigen or the orientation of the *cer *fragment considered. One should note that the nature of antigen itself has some influence on the maintenance of the plasmid. In the case of Urease, the stability decrease is more important, for both vectors, and once again more marked for the vector devoid of *cer *fragment as compared to the vector containing *cer*, whatever the orientation considered. Dramatic differences are already observed at time points between 2 and 6 hours.

**Table 1:** Example of *Helicobacter pylori *AlpA protein produced in erlen flask in absence of Kanamycin

Culture time	Plasmid without *cer*	With *cer *orientation1	With *cer *orientation2
2 h(IPTG added)	97%	100%	100%

4 h	25%	100%	100%

6 h	20%	75%	100%

23 h	3%	79%	97%

The contribution of the *cer *fragment in increasing the plasmid maintenance over time is clearly established, a slight difference is observed according to the relative orientation of *cer *but should be confirmed by additional experiments

**Table 2:** Example of *Helicobacter pylori *Urease produced in erlen flask in absence of kanamycin

Culture time	Plasmid without *cer*	With *cer *orientation1	With *cer *orientation2
1 h	87%	100%	100%

2 h	IPTG addition		

3 h	67%	100%	100%

5 h	1%	50%	34%

25 h	0%	9%	8%

The contribution of the *cer *fragment in increasing the plasmid maintenance over time is again clearly established with a different antigen, the difference observed according to the relative orientation of *cer *is in the opposite direction as compared to AlpA and likely to be non-significative.

The real value of the 24 hours end points is matter of discussion since one can argue that an expression driven by the T7 promoter is likely to cause a certain level of metabolic breakdown of the cellular system and make questionable a cultivation experiment for more than 25 hours post induction. Even if protein and/or induction condition dependent, this is perfectly true but nevertheless important to document plasmid stability at end and after normal end of fermentation to document regulatory files.

We have demonstrated, or at least re-demonstrated that an appropriate stabilisation of the expression vector is a first step towards the suppression of antibiotic selection during culture.

### Second step: evaluation of the poison/antidote system in fermentation conditions

The original poison/antidote system was chosen for different reasons:

- the host/vector couple is commercially available from Delphigenetics,

- the engineered strain is a BL21λ(DE3) derivative, making comparison with our standard process easier.

In a pharmaceutical context, the presence of minute amount of ccdB as contaminant would be acceptable, since no equivalent of gyrase is present in human cells. The experiment was designed in order to show whether this antibiotic-free selection would be applicable in fermentation at 1 liter and larger scale.

The model system was based on the pM1816 vector used for the production of recombinant Pseudomonas exotoxin (rEPA), a pM1800 derivative vector containing the kanamicyn resistance gene and the *cer *fragment. The *ccdA *encoding gene extracted from the pStaby cloning vector obtained from Delphigenetics was cloned into the pM1816 expression vector generating the pSP1 construct. In order to eliminate the Kanamycin resistance gene a digestion with ClaI and AscI restriction enzymes followed by self-religation has been done.

Fermentation at 1 liter scale (table 3) was done in absence of Kanamycin for pM1816 and 2 different induction protocols were applied:

**Table 3:** Evaluation of antibiotic-free system in fermenters (production of rEPA protein)

	Induction at early stage of growth		Induction at advanced stage of growth	
	**System based on kana resistance**	**Antibiotic free system**	**System based on kana resistance**	**Antibiotic free system**

Cell dry weigh (g/L)	28	23	22	24

Plasmid retention (%)	5	100	90	98

Product yield (mg/L)	36	350	280	603

Specific productivity (mg product/gCDW)	1	15	13	25

Two conditions have been analyzed: induction at A_600 _= 25 (advanced stage of growth), close to standard production, and induction at A_600 _= 1 (early stage of growth) to increase the number of cell division between induction and end of fermentation and introduce an extended high level of stress over the cells.

In normal conditions the plasmid maintenance is increased and productivity doubles. For extended stress condition, the differences between antibiotic-free and standard are dramatic, both in terms of plasmid maintenance and protein productivity.

- a standard induction at high cell density (A_600 _= 25),

- an early induction (A_600 _= 1), not representative of normal fermentation conditions, to increase the number of cell divisions between induction and end of fermentation and thus artificially creating extended high stress conditions. In that case the higher stress would be due to high metabolic charge to ensure multiple cell divisions in parallel to protein production. It is likely to say that in such condition the probability to lose plasmid would be greater than using induction at high cell density. The 3 following parameters have been monitored at the end of fermentation:

- absorbance,

- percentage of cells still containing a plasmid,

- amount of recombinant protein expressed.

**Figure 1:**
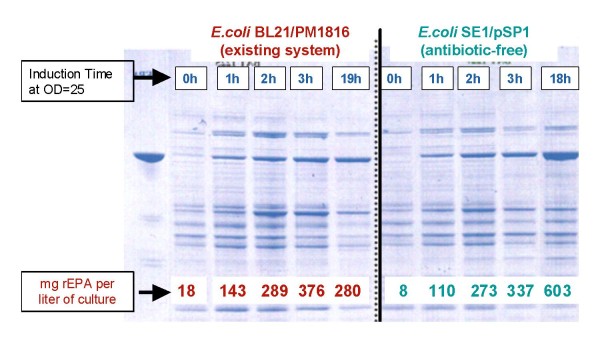
**protein production evaluation in 1 liter fermenter**. Upon induction, the behaviour of both systems is comparable but a clear increase in protein production is observed with the antibiotic-free system at the end of fermentation.

When standard fermentation conditions (A_600 _= 25) are applied, the plasmid maintenance is at least equivalent or superior when the poison antidote selection is compared to the standard vector (98% versus 90%). The unanticipated result is that the amount of expressed antigen is two fold increased with the poison antidote system (603 mg/L versus 280 mg/L)(Figure 1). This is possibly due to a more complete elimination of bacteria devoid of plasmid and thus preferable maintaining of the hosts producing the recombinant protein of interest. In the context of the experiment described, the antibiotic-resistance gene was eliminated through cut and re-ligation leading to another possible and complementary explanation. One can argue that the kanamycin resistance gene, if present, even in absence of antibiotic pressure, would be transcribed and translated and be responsible for an additional stress on the cell machinery and detrimental to the global expression of recombinant antigen. We can assume that the transcription and translation of *ccdA*, which is a small molecule, would have a minor overloading effect on the cell machinery.

**Figure 2:**
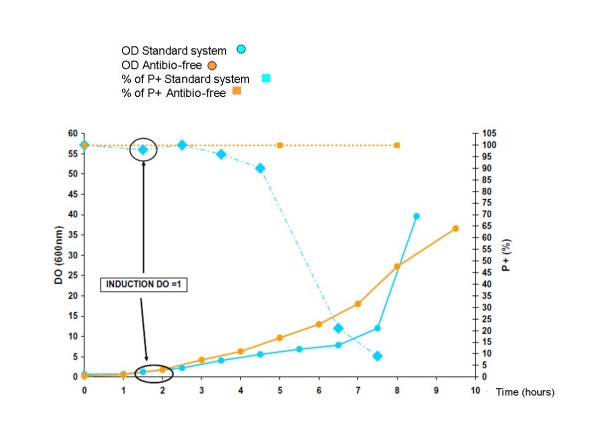
**Follow-up of Plasmid stability and Optical density for cultures in fermenter (1 litre scale) upon induction at early stage of growth**. As early as 2 hours after induction the % of P+ cells start decreasing and drops dramatically after 4 hours for the standard system. Virtually no decreasing effect is observed with the antibiotic-free selection in the same conditions.

In artificial "extended high stress" conditions (A_600 _= 1, Table 3 and Figure 2), the plasmid loss is extremely rapid with the standard vector as compared to poison/antidote (5% versus 100%), indicating the very stringent selection of the ccdA/ccdB system. The 10 fold difference in the amount of expressed antigen is fully consistent with the observed difference in term of plasmid maintenance (36 mg/L versus 350 mg/L).

In order to evaluate the potential for scale-up, the fermentation process previously established, for the standard conditions, was transposed at a 30 liters scale. As indicated in table 4, values obtained for both optical densities at different times and plasmid maintenance is almost similar.

**Table 4:** Scale-up of antibiotic-free system

Fermenter scale	1 L		30 L	
Induction time	3 h	20 h	3 h	20 h

A_600_	72	47	73	55

P+ %	100	98	100	100

rEPA (mg/L)	337	603	194	275

No marked differences appear between 1 liter and 30 liters fermentation for the antibiotic-free system in terms of both, cell density and % of P+ cells.

### Third step: possible contribution of the *cer *locus

In order to evaluate the real contribution of the *cer *locus on the observed increased expression level, a new series of vectors capable to express the recombinant AlpA protein have been assembled and tested: pM-Hp3.1 (*cer *-, KanR +, *ccdA-*), pSP3 (cer-, KanR-, *ccdA *+) and pSP5 (*cer *+, KanR-, *ccdA*+) (table 5).

**Table 5:** Summary of plasmids used

Plasmid name	Derived from plasmid	Recombinant protein expressed	F1 origin	*cer *fragment	Kanamycin resistance gene	*ccdA *gene
**pET28c**		-	+	-	+	-

**pM1800**	pET28c	-	-	+	+	-

**pMH.P3.1**	pET28c	AlpA	+	-	+	-

**pM1816**	pM1800	rEPA	-	+	+	-

**pSP301**	pM1800	-	-	+	Rcb	+

**pSP1**	pM1816	rEPA	-	+	-	+

**pSP2**	pSP301	-	-	-	Rcb	+

**pSP3**	pSP2	AlpA	-	-	Rcb	+

**pSP4**	pSP2	rEPA	-	-	Rcb	+

**pSP5**	pSP301	AlpA	-	+	Rcb	+

**pSP6**	pSP301	rEPA	-	+	Rcb	+

-: absent; +: present; Rcb: kanamycin resistance gene is deleted through homologous recombination

**Figure 3:**
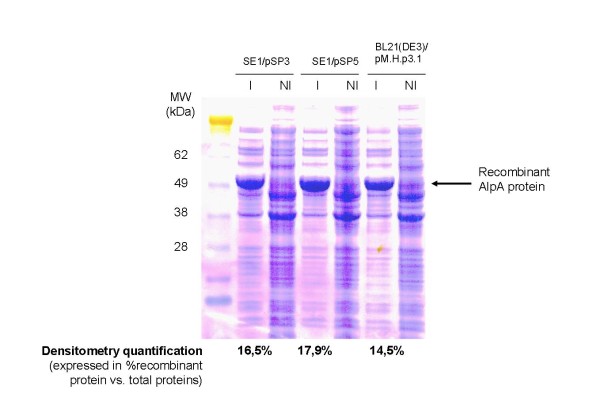
**Head to head comparison of the expression of the same antigen with 3 different vectors**. *Helicobacter pylori *AlpA protein was produced in erlen flask in absence of Kanamycin using three differents vectors: pM.H.P3.1 (control vector -*cer*), pSP3 (antibiotic-free vector -*cer*) and pSP5 (antibiotic-free vector +*cer*). Total lysates of induced and non induced bacteria were loaded on SDS-PAGE and a densitometry quantification of the recombinant protein was performed using GeneTools software. This quantification is expressed as a percentage of the protein of interest compared to total proteins. The results show the contribution of both antibiotic-free system and *cer *locus in protein expression improvement (antibio-free + *cer *> antibio-free - *cer *> standard - *cer*).

A head to head comparison of protein of interest versus total proteins on a SDS-PAGE using densitometry quantification (GeneTools software) clearly shows that for a given antigen (figure 3):

- antibiotic-free selection - *cer *locus gives superior production level (16,5%) compared to standard antibiotic selection - *cer *(14,5%).

- antibiotic-free selection + *cer *gives also superior production (17,9%) compared to the same construct devoid of *cer *locus (16,5%).

The following hierarchy can be established:

antibio-free + *cer *> antibio-free - *cer *> standard - *cer*

Even if limited to a single antigen, the experiment shows a benefit, in terms of protein yield, by combining the antibiotic-free selection and the stabilizing element.

### Final step: elimination of the antibiotic resistance gene

Antibiotic selection and therefore, the presence of an antibiotic resistance gene on the vector until the last step of the expression are required to ensure the absence of external contaminant cells that would not be counter-selected by the poison/antidote system.

Even if antibiotic selection pressure is not used during the fermentation process, removal of this antibiotic resistance marker is of major importance to prevent horizontal transfer in the environment. This is particularly true for vectors to be used in gene therapy or DNA vaccination protocols. For above mentioned reasons (see second step) it is also important to eliminate this sequence to improve both vector stability and recombinant protein expression level.

Elimination of the antibiotic resistance marker can be very simply obtained by restriction enzyme digestion using unique sites flanking the sequence to be removed followed by self-ligation of the vector. The only issue being that the discrimination between correctly deleted and parental vectors is not directly possible and would involve time consuming analytical techniques such as, restriction mapping following a small scale DNA preparation or alternatively a PCR-based approach.

**Figure 4:**
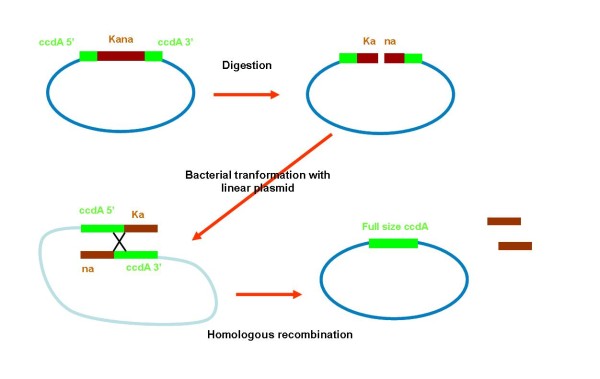
**Homologous recombination process allowing assembly of a functional *ccdA *encoding gene**. After transformation of SE1 bacteria expressing the *ccdB *gene product with linearized plasmid DNA, a functional *ccdA *gene is assembled through homologous recombination. The expression of the *ccdA *gene product allows survival of the recipient strain and provides a positive selection ensuring the elimination of the Kanamycine resitance gene.

**Figure 5:**
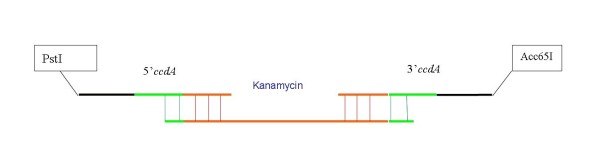
**Construction by combined PCR of the 5'*ccdA*-Kanamycin-3'*ccdA *fragment**. In green the 120 bp sequence identical in 5'*ccdA *and 3' *ccdA *fragment and target sites for homologous recombination, in blue the kanamycin resistance gene. Homologous recombination ensures the complete elimination of the Kanamycin resistance gene.

In order to overcome the problem we have designed a model, including a positive selection, in which the antibiotic-free selection marker would be functional only after proper elimination of the antibiotic resistance gene. In this model (figure 4 and 5) the *ccdA *locus is split into 2 parts, containing a common sequence, and cloned in the 5' and 3' regions flanking the antibiotic resistance gene. After digestion at a unique restriction site located inside the antibiotic selection marker and transformation of ccdB expressing cells with linear DNA, a fully functional *ccdA *would assemble through homologous recombination. Only CYS21 and SE1 bacteria containing a recombinant plasmid with a functional *ccdA *can grow upon transformation.

### Recombinant protein production and/or Plasmid DNA preparation

The above-described work is deliberately focused on recombinant protein production, for which the advantage of combining a stabilisation element and an antibiotic-free selection mean has been illustrated. It would be easy to extrapolate and say that a system allowing 100% maintenance of plasmid during fermentation would also give superior results in terms of amount of plasmid recovery. We have done some preliminary experiments towards that direction (data not shown). The results observed are a tendency to obtain 10 to 20% more plasmid with the antibio-free + *cer *plasmid. But, only a repetition of large series of experiments would allow us to be really confident on that observation, for the following reasons:

- The techniques for midi or maxi plasmid DNA extraction suffer from a certain batch to batch variability (at least at lab scale),

- A real head to head comparison is difficult, since the comparison has to be done with non-identical bacterial strains (CYS21 vs DH10B in our case).

Nevertheless, we would say that, in addition to a potential superiority in production, the real added value of our system to plasmid DNA preparation is the one-step selective elimination of the antibiotic resistance gene that is mandatory for therapeutic applications.

## Conclusions

Antibiotic-free selection is a general and ultimate goal that can be attained by the implementation of various and combined approaches. An increasing knowledge of bacterial physiology will give access to more and more essential genes or pathways that would be an unlimited source of inspiration for the design of novel selection means. The major driver for the definition of antibiotic-free systems is a desire to anticipate and fulfil future recommendations from health authorities to overcome safety concerns but, interestingly it can give access to unexpected properties such as a marked increase in recombinant protein production or plasmid recovery. The complete elimination of any antibiotic resistance gene is, for different reasons, of critical importance for both recombinant protein production and DNA immunisation vectors. The proposed homologous recombination process has been demonstrated to be fully functional and might be associated to different antibiotic-free expression systems or transferred to other microorganisms.

## Methods

### Vector containing the *cer *fragment: pM1800 and its derivatives

Table 5 summarizes the properties of mentioned plasmids.

The pET28c vector (Novagen) was amplified by PCR using the « expand long template PCR system » kit from Boehringer Mannheim in order to suppress the *f1 *origin.

The 5' primer carried the site AscI and the 3' primer, AscI and PacI. After amplification, the PCR product was purified, AscI (New England Biolabs) digested and self-ligated with T4 DNA ligase, (New England Biolabs).

A 300 bp fragment containing the sequence of *cer *fragment was obtained by PCR amplification using the pXL 2979 plasmid (gift of Rhône Poulenc) as matrix. After purification, the PCR product was digested by AscI and PacI (New England Biolabs), and was cloned into pET28c devoid of *f1 *origin previously obtained digested by these same enzymes. This new plasmid was termed **pM1800**.

The same strategy has been used to obtain the same plasmid with a *cer *fragment in the opposite orientation.

**Figure 6:**
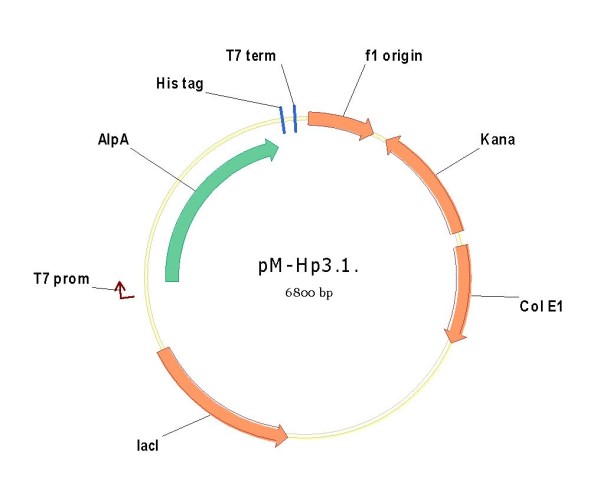
**Plasmid pM-Hp 3.1, derivative of pET28, original construct expressing the *Helicobacter pylori *AlpA antigen**.

To express AlpA protein, the *H. pylori *X47-2 strain was used as a source of DNA. A 1.6 kb DNA fragment containing the sequence encoding the mature AlpA protein was amplified by PCR and cloned into pM1800 between the NcoI and XhoI restriction sites, yielding the **pM-Hp3.1**.(figure 6)

To express the recombinant exoprotein A from *Pseudomonas aeruginosa *(rEPA), the plasmid pVC45 D (an Ampicillin resistant plasmid provided by Temple University, USA) containing the recombinant *exoprotein A *(*rEPA*) gene cloned in fusion with the *OmpA *signal sequence between HindIII and EcoRI sites has been used. To avoid a PCR step, this plasmid was digested by XbaI (New England Biolabs) and EcoRI (New England Biolabs) to isolate the fragment containing the RBS sequence and the OmpA-rEPA fusion (2024 bp). After purification, the fragment was cloned into plasmid pM1800 digested by the same enzymes to create plasmid **pM1816 **(7195 bp).

### Vector containing the *ccdA *fragment: pSP301, pSP1, pSP2 and their derivatives

**Figure 7:**
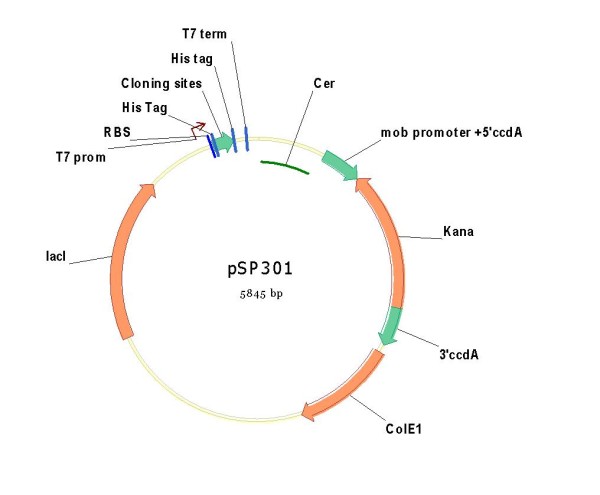
**Plasmid pSP301 containing a Kanamycin resistance gene flanked by 5' and 3' parts of the *ccdA *gene (non functional)**.

First the pM1800 has been amplified by a PCR reaction to eliminate the kanamycin resistance gene and to create the PstI and Acc65I sites. In the same way the fragment containing the *mob *promoter, the 5' part of *ccdA*, the kanamycin resistance gene and the 3' part of *ccdA *gene (figure 5) was constructed by combining and extending PCR amplicons. Both, the 5' *ccdA *and 3' *ccdA *fragments contain the same 120 bp sequence. Finally, the PCR product was digested by PstI (New England Biolabs) and Acc65I (New England Biolabs) and cloned in the pM1800 devoid kanamycin resistance gene. The new plasmid was termed **pSP301 **(figure 7).

Then, pSP301 vector was digested by PacI (New England Biolabs) and AscI (New England Biolabs) to eliminate the *cer *fragment. The cohesive ends were digested by the Mung Bean exonuclease (New England Biolabs), and the plasmid was self- ligated to create the new vector **pSP2**.

**Figure 8:**
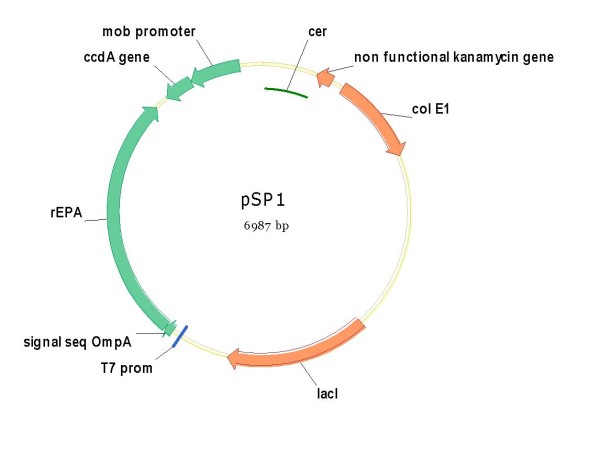
**Plasmid pSP1 containing the functional *ccdA *cassette, the *cer *fragment and an almost completely truncated kanamycin resistance gene**.

The pM1816 plasmid has been also modified to express the rEPA protein and *ccdA*. The *mob *promoter and the *ccdA *gene were amplified from the pStaby (Delphigentics) and cloned into pM1816 between the site Not I and EcoR I. To eliminate the Kanamycin resistance gene, this new plasmid pM1816+*ccdA *was digested by ClaI (New England Biolabs) and AscI (New England Biolabs). The cohesive ends were digested by the Mung Bean exonuclease (New England Biolabs), and the plasmid was self-ligated to create the new vector **pSP1 **(figure 8).

**Figure 9:**
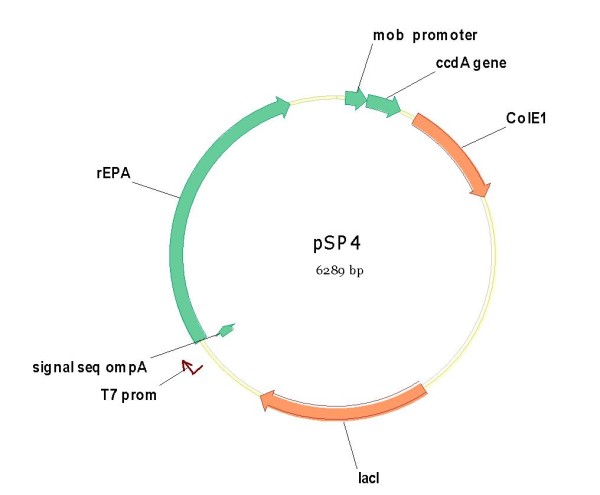
**Plasmid pSP4, similar to pSP1 but devoid of *cer *fragment**.

**Figure 10:**
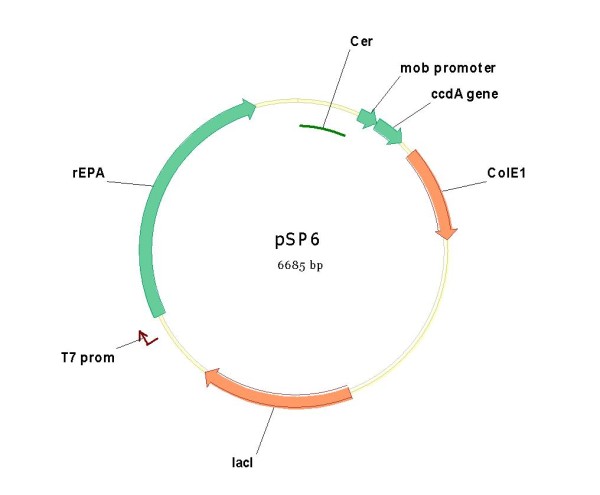
**Plasmid pSP6, similar to pSP1 but completely devoid of Kanamycin resistance gene**.

The XbaI-EcoRI fragment containing the RBS sequence and the OmpA-rEPA fusion, isolated from this pM1816 vector was cloned into both pSP2 and pSP301 between the same unique restriction sites. The kanamycin resistance gene of these new plasmids (pSP2+ rEPA; pSP301+rEPA) was eliminated by recombination yielding **pSP4 **and **pSP6 **respectively (figures 9 and 10).

**Figure 11:**
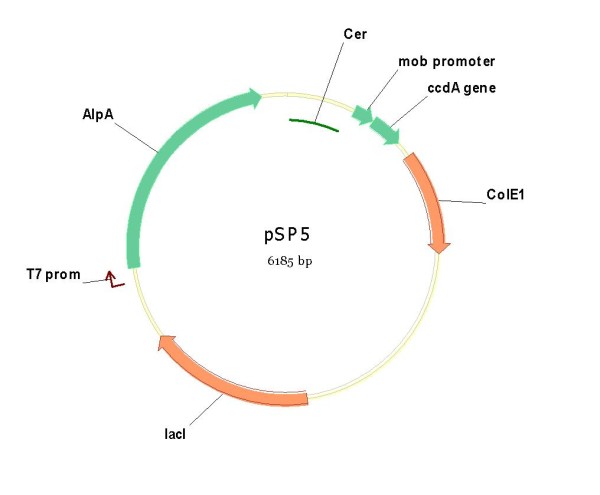
**Plasmid pSP5 containing the *cer *fragment, a functional *ccdA *gene and the expression cassette for the *Helicobacter pylori *AlpA antigen**.

For AlpA the same strategy has been used. The XbaI-XhoI fragment containing the sequence of the mature AlpA isolated from the pM-Hp3.1 vector, was cloned into both pSP2 and pSP301 between the same unique restriction sites. The kanamycin resistance gene of these new plasmids (pSP2+ AlpA; pSP301+AlpA) was eliminated by recombination yielding **pSP3 **and **pSP5 **respectively (figure 11).

### Homologous recombination process to obtain a functional *ccdA*

To use the ccdA/ccdB selection system, the Kanamycin resistance gene was eliminated through homologous recombination. The pSP301 and derived plasmids were digested by ClaI (New England Biolabs). The CYS21 strain (DH10B derived with *ccdB *in the chromosome) (Delphigenetics) was transformed with the linear plasmid, and plated on LB agar. Only the clones containing a correctly recombined plasmid encoding a functional *ccdA *gene could grow. The presence of *ccdA *was checked by enzymatic digestion and sequencing.

Cla I is a unique site in the kanamycin resistance gene, and EcoR V is unique in the vector. When the vector contains the Kanamycin resistance gene two bands are present for the ClaI-EcoRV digestion. If the recombination event behaves as expected and eliminates the kanamycin resistance gene, only one band is likely to be present for the ClaI-EcoRV digestion (data not shown).

### Expression at lab scale

To express the recombinant proteins the SE1 bacteria derived from BL21λ(DE3) (with *ccdB *in the chromosome) were transformed with pSP1, pSP3, pSP4, pSP5 and pSP6, and plated on LB agar. Similarly, the BL21λ(DE3) strain (Invitrogen) was transformed with the PM1816 or pM-Hp3.1 and plated on LB kanamycin (25 μg/ml). An overnight culture for each construction was diluted 1/100 in 50 ml of fresh LB with or without Kanamycin. All cultures were grown at 37°C to an A_600 _= 0.4-0.6. The cultures were divided in 2 × 25 ml and the expression of the recombinant exoprotein A was induced using 1 mM Isopropyl-β-D-thiogalactopyranoside (IPTG) in one of 25 ml culture. The induced and non-induced bacteria were grown at 37°C up to 25 H post induction.

### Evaluation in fermenters

The comparison at bench scale of the new system with the existing system based on the kanamycin resistance involves studies of plasmid retention, product yield and robustness.

The cultures at lab scale were conducted with the Biostat Q BBi Sartorius system which includes 4 individual fermentors of 500 ml as working volume.

The culture at pilot scale was conducted in a Applikon fermentor of 30 liters as working volume. This is the current scale for clinical batches for a phase 1 study.

The medium used is a "in house" propietary complex medium (Yeast extract as source of nitrogen and dextrose as source of carbon) without the use of antibiotics.

Cultures were regularly sampled to monitor the in-process parameters such as A_600_, Cell Dry Weight (CDW) (g/L), numeration (CFU/ml), product yield and plasmid retention (%)

#### Product yield

Each sample of cultures was treated by osmotic shock to release the periplasmic content. Following this, each extract was titrated by ELISA and loaded on SDS-PAGE. A densitometry quantification has been done using GeneTools software to compare the protein of interest versus total proteins.

### Plasmid stability monitoring

Samples of cultures were diluted and then, plated on solid media for the numeration

For the system based on the kanamycin resistance, 100 individual colonies were picked on LB agar supplemented with kanamycin (25 μg/mL).

For the antibiotic free system, 96 colonies were picked to inoculate individual liquid cultures in microplates. After 24 h of incubation, the plate was centrifuged and then placed into a robotic pDNA extraction system (QIAgen bioroBot^® ^System). Finally, the samples were loaded on 1% agarose gel to detect the presence or absence of plasmid.

The readout of the stability is provided by a simple numeration of colonies still containing the plasmid as a percentage of the total analyzed colonies.

## Competing interests

No sources of funding were used to assist in the preparation of this article. The authors have no conflict of interest directly relevant to the content of this article.

## Authors' contributions

IP carried out the molecular biologic constructs strategy, NC and CM carried out the fermentation studies, GH and SH participated to the molecular biology part, VC coordinated the fermentation studies, KD participated in drafting the manuscript, DS and RS designed and coordinated all the study. All authors read and approved the final manuscript.
